# Patient Perceptions of Traditional Bonesetters and Determinants of Their Utilization: A Cross‐Sectional Study in Iraqi Kurdistan

**DOI:** 10.1002/hsr2.71935

**Published:** 2026-02-28

**Authors:** Rukhsar Muhammad Omar, Salar Omer Abdulqadir, Sirwan Khalid Ahmed, Moustaq Karim Khan Rony

**Affiliations:** ^1^ Department of Kindergarten, College of Basic Education University of Raparin Sulaymaniyah Kurdistan Region Iraq; ^2^ College of Nursing University of Raparin Sulaymaniyah Kurdistan Region Iraq; ^3^ Miyan Research Institute International University of Business Agriculture and Technology Dhaka Bangladesh

**Keywords:** bonesetter, Iraqi Kurdistan, orthopedic care, patient perception, traditional treatment

## Abstract

**Background and Aims:**

Despite the prevalent reliance on traditional bonesetters (TBSs) in Kurdish societies, their role remains underexplored in scholarly research. This study aimed to evaluate patients' perceptions of TBSs and to investigate the determinants of their utilization over modern orthopedic care.

**Methods:**

A cross‐sectional study was conducted from August 20 to September 20, 2022, using purposive sampling. A structured questionnaire, adapted from prior studies and validated (Cronbach's *α* = 0.73), assessed sociodemographic characteristics, healthcare experiences, and perceptions. *χ* ^2^ test and multivariate logistic regression analysis were applied to identify factors associated with perspectives toward TBSs.

**Results:**

The study included 106 participants. The majority of participants were aged 18–45 (62%), 51.9% male, and 61.3% urban residents. Perceptions toward TBSs were 34.9% positive, 34.9% negative, and 30.2% neutral. The mean perception score was 4.80 ± 1.40, with 5 as the cutoff for neutral perception. Multivariate analysis revealed that disagreement with hospital staff attitudes (AOR = 0.04, 95% CI: 0.07–0.23, *p* < 0.001) and fear of hospital procedures (AOR = 0.13, 95% CI: 0.02–0.85, *p* = 0.033) were significant predictors of TBS preference, while financial constraints and cultural factors were not significant.

**Conclusion:**

The continued reliance on TBSs in Northern Iraq highlights a critical gap in patient trust toward formal healthcare systems, particularly regarding hospital staff interactions and procedural concerns. Addressing hospital staff attitudes and improving patient confidence in modern medical procedures may help shift preferences toward evidence‐based orthopedic care.

## 1 Introduction

Traditional healing practices remain prevalent worldwide and have been recognized by numerous scientific institutions. The World Health Organization (WHO) has emphasized in its Traditional Medicine Strategy that traditional medical practices continue to evolve globally [1]. Integrating traditional bonesetters (TBSs) with modern orthopedic care presents an opportunity to assess the strengths and limitations of both approaches, thereby leveraging cultural competencies in medical treatment [2].

Despite substantial advancements in orthopedic medicine and fracture management, many individuals continue to rely on TBSs. This preference is strongly influenced by cultural and religious beliefs, economic factors, and accessibility [3, 6]. The affordability and immediate availability of TBSs further contribute to their popularity [4]. Even in developing regions such as Africa, Asia, and South America, TBSs remain widely utilized for the treatment of fractures and musculoskeletal injuries ([5]; Onyemaechi [6, 7]). For instance, in some parts of Nigeria, TBSs manage 70%–90% of all musculoskeletal injuries [8], while 60% of trauma patients in India [9] and over 50% in Tanzania [10] seek their services.

Although TBSs play a significant role in healthcare, their practice faces substantial criticism and legal challenges (Onyemaechi [11]). Most TBSs acquire their skills through family traditions rather than formal education, leading to a lack of knowledge regarding infection control, bone healing principles, and patient safety (Onyemaechi [6]). This knowledge gap increases the risk of infection, malunion, and nonunion fractures, and in some cases, aggressive manipulation by TBSs may exacerbate injuries [10]. Furthermore, due to inadequate training in anatomy, physiology, and hygiene protocols, the risk of complications and disease transmission among patients treated by TBSs remains high [12]. However, collaboration between TBSs and orthopedic surgeons could potentially enhance patient outcomes. By participating in formal training programs and integrating modern medical principles, TBSs may improve treatment efficacy and reduce complications [4, 13]. Establishing formal regulatory institutions to connect TBSs with healthcare professionals could enhance patient safety and healthcare coordination [14].

In Kurdistan, Northern Iraq, there is limited research on traditional medicine and no official data on the number of TBSs, their treatment methods, or patient outcomes. TBSs have historically played a critical role in primary healthcare, particularly in rural and underserved areas. One of the distinguishing features of TBSs is their reliance on ancient healing techniques [15]. People continue to seek TBSs' services due to their accessibility, familiarity, and alignment with cultural traditions [2]. This study aims to explore patient perspectives on TBSs and the factors influencing their utilization in Kurdistan, Northern Iraq. Understanding these factors could inform healthcare policies and interventions aimed at enhancing patient safety and treatment outcomes.

## 2 Methods

### 2.1 Study Design, Setting, Period and Eligibility Criteria

This study employed a cross‐sectional research design to examine perspectives on traditional bonesetters (TBSs) and the factors influencing their utilization in Kurdistan, Northern Iraq. The study was conducted across various regions within the Kurdistan Region, ensuring the inclusion of diverse demographic and geographic representations. The research targeted individuals aged 18 years and older, as this age group is expected to have sufficient awareness and decision‐making capacity regarding healthcare choices. Data collection was conducted through structured face‐to‐face interviews with participants using a validated questionnaire. The study adopted a non‐probability purposive sampling method to select individuals who had prior experience with TBSs. While this approach allowed for targeted recruitment of participants with relevant experience, it may have introduced selection bias and limited the generalizability of the findings. Future research should consider employing probability‐based random sampling to ensure that the sample more broadly represents the target population. The recruitment strategy was designed to ensure representation from both rural and urban populations, capturing a broad spectrum of perspectives. Data collection took place over a 1‐month period, from August 20 to September 20, 2022. To be eligible for participation, individuals had to express a willingness to participate, demonstrate cognitive and psychological stability to provide reliable responses, and have firsthand experience with TBSs. Participants under the age of 18 were excluded due to ethical considerations and concerns regarding their ability to provide informed consent.

### 2.2 Study Tools

The study utilized a structured questionnaire, which was adapted and modified from previously validated studies [3, 4, 16] to ensure cultural and contextual relevance to the Kurdish population. The questionnaire was comprehensive and divided into four major sections to capture relevant information systematically. The first section focused on sociodemographic characteristics, including age, gender, marital status, educational background, occupation, financial status, and residential area. Additionally, participants were asked about their awareness of TBSs and the sources from which they obtained information regarding TBS services. The second section assessed factors influencing participants' decisions to seek treatment from TBSs, covering economic constraints, dissatisfaction with hospital‐based care, cultural beliefs, prior experiences with both TBSs and orthopedic specialists, and fear of hospital procedures such as surgery or amputation. The third section explored participants' perceptions toward TBSs, including their level of trust in TBSs, belief in the competency of TBSs compared to orthopedic specialists, and perceptions regarding the quality of patient care provided by TBSs. Participants were also asked about their opinions on the potential collaboration between TBSs and formal healthcare providers. The fourth section comprised interviews with traditional bonesetters, which provided deeper insights into their training background, treatment approaches, frequency of patient visits, types of conditions treated, and experiences with patient complications. Each interview lasted approximately 40 min and was structured to explore the extent of their medical knowledge, treatment methodologies, and perspectives on formal medical training.

To ensure content validity and cultural appropriateness, the questionnaire was subjected to a pilot study involving 10 participants. The internal consistency of the perceptual assessment items was evaluated using Cronbach's alpha, yielding a reliability coefficient of 0.73, indicating acceptable internal consistency [17].

### 2.3 Study Measures

The primary outcome variable of this study was participants' perspectives toward TBSs, assessed using a seven‐item scale. Responses were recorded in a binary format (“Yes” or “No”), where a “Yes” response was assigned a score of 1 and a “No” response was assigned a score of 0. The total score ranged from 0 to 7, which was further categorized into three perception levels:

- Negative perception (0–4 points)
- Neutral perception (5 points)
- Positive perception (6–7 points)

In addition to the primary outcome variable, several independent variables were examined, including age, gender, marital status, educational attainment, occupation, financial status, and place of residence. Furthermore, variables related to medical history, such as prior experience with fractures, types of fractures, and history of orthopedic consultations, were also analyzed.

### 2.4 Ethical Approval and Inform Consent

This study was conducted in accordance with the Institutional Research Ethics Board and the Declaration of Helsinki to uphold ethical integrity and participant rights. The research received ethical approval from the Scientific Review Committee of the University of Raparin, College of Nursing (Ref: 7/29/3721).

Before participation, all respondents were provided with detailed information about the study's objectives, procedures, and potential implications. Participants were required to sign an informed consent form, affirming their voluntary participation and understanding of the study. Confidentiality and anonymity were strictly maintained, with all personal data securely stored and used exclusively for research purposes. Participants were granted the right to withdraw at any stage without consequence.

To ensure the ethical treatment of traditional bonesetters, those interviewed were informed about the nature of the research and their consent was obtained prior to data collection. Researchers also ensured that no coercion or undue influence was applied to any participant.

### 2.5 Statistical Analysis

Data were coded, entered, and analyzed using IBM SPSS Statistics version 25. Descriptive statistics, including frequencies and percentages, were used to summarize categorical variables, whereas means and standard deviations were computed for continuous variables. The Shapiro–Wilk test and histogram visualization were employed to assess the normality of data distribution, confirming that all study variables followed a normal distribution. To examine associations between sociodemographic characteristics and perspectives toward TBSs, the *χ* ^2^ test was conducted. Variables that showed a significance level of *p* < 0.25 in the univariate analysis were further tested in a multivariate logistic regression model to identify independent predictors of positive perspectives toward TBSs. The results of the multivariate regression analysis were reported using adjusted odds ratios (AOR) with 95% confidence intervals (CI). A *p*‐value of < 0.05 was considered statistically significant, indicating strong evidence of an association between specific factors and positive perceptions of TBSs.

## 3 Results

### 3.1 Characteristics of the TBSs

In the present study, two male traditional bonesetters (TBSs) were interviewed, each with extensive experience in the field. The first TBS was 56 years old, with 30 years of professional experience, and had only completed primary school education. He acquired his skills through self‐exploration without formal instruction. His practice operates 5 days a week, receiving approximately 40 patients per day, including individuals from outside his province. Patients seek his services for a range of musculoskeletal conditions, including fractures, vertebral issues, and nerve‐related complications. His treatment methods include massage therapy, traction, joint manipulation, and traditional casting techniques. He primarily utilizes cardboard and wooden splints for fracture fixation and administers nonsteroidal anti‐inflammatory drugs (NSAIDs), multivitamins, and vapor‐coolant sprays. Importantly, he does not charge fixed fees but allows patients to contribute voluntarily. Additionally, he refuses to treat patients with open fractures, severe injuries requiring surgery, or cases that necessitate orthopedic intervention. Notably, he reported no prior legal disputes or major complications related to his practice.

The second TBS, aged 46 years, had 15 years of experience and had completed high school education. Unlike the first bonesetter, he acquired his expertise from his father rather than through self‐teaching. His practice is conducted from home rather than a dedicated clinic, and he treats 5–10 patients per day. His scope of practice includes joint dislocations, fractures, sprains, strains, and knee ligament injuries. His techniques involve massage therapy, traction, joint adjustments, and traditional casting. Unlike conventional orthopedic plaster casts, he employs herbal compounds such as The Gana Garchak tree (Botanical name: *Ailanthus altissima*), which is traditionally believed to possess analgesic properties when applied topically, while onion (*Allium cepa*) and eggplant (*Solanum melongena*) peels are used in folk medicine for their purported anti‐inflammatory effects. Nylon wraps are applied tightly around affected areas to reduce muscle spasms, functioning similarly to compression therapy. These methods, though widely practiced among TBSs, lack formal clinical validation and vary in preparation and application techniques. Similar to the first bonesetter, he does not charge patients and refuses to treat complex fractures such as comminuted, spiral, or compound fractures, as well as patients with chronic conditions like diabetes, coronary artery disease, or osteoporosis. He requests diagnostic imaging (X‐rays, CT scans, or MRIs) for obese patients whose fractures cannot be manually detected. He also reported no legal disputes or major complications in his career (Table 1).

**Table 1:** Characteristics of the TBSs.

Bonesetters	Age	Work experience	Level of education	acquired the skill	Number of patients every day	kinds of problems	Place of work	kinds of procedures and techniques	Tools used	Amount of charge	Type of patient	Type of pharmacological compounds
First	56	30 years	Primary school	Personal exploration without a teacher	40	Fractures, crakes, vertebral problems	At clinic	Massage therapy, traction, casting joint replacement	Cartoon and a piece of wood for fixing the fractured part	Patient's preference	He won't receive a patient with open wounds or someone who needs surgery or he feels that the patient needs to meet orthopedics	Multivitamins, NSAIDs and vapor coolant sprays
Second	46	20 years	High school	Acquired the experience from his father	15	Joint dislocations, nervous system problems, fractures, crakes, sprains, strains, vertebral and knee ligament problems	He does not have a clinic and receives patients at his home.	Massage therapy, traction, casting joint replacement	Herbal compounds such as Gana Garchak, tree leaves as painkillers, nylon for the site of spasm, and eggplant leaf peel, and onion peels as an anti‐inflammatory treatment. He uses raw cloth with wood for fixing and casting a fractured site instead of plaster	No charge	He does not work for patients with comminuted, spiral or compound fractures or patients with chronic diseases like diabetics, coronary artery disease or patients with osteoporosis and referred them to the orthopedics	He uses some herbal compounds such as Gana Garchak tree leaves as painkillers, nylon for the site of spasm, and eggplant leaf peel, and onion peels as an anti‐inflammatory treatment

### 3.2 Sociodemographic Characteristics of Clients

The study enrolled 106 participants, of whom 51.9% were male and 48.1% were female. The majority (62%) were aged between 18 and 45 years, a range typically associated with increased risk of fractures due to occupational hazards and traffic accidents. Marital status distribution indicated that 72.6% were married, 19.8% were single, and 7.5% were widowed or divorced. In terms of educational background, 32.1% had completed secondary school, 27.4% were illiterate, 21.7% had only completed primary school, and 8.5% had attained a bachelor's degree or higher. Regarding employment status, 39.6% were self‐employed, 25.5% were government employees, and 24.5% were housewives. The majority of participants (61.3%) resided in urban areas, whereas 26.4% lived in suburban regions, and 12.3% were from rural communities. Financial assessment revealed that 61.3% had sufficient income, 23.6% had barely sufficient income, and 15.1% faced financial difficulties. The primary source of information about TBSs was relatives (56.6%), followed by friends (9.4%), social media (1.9%), and other sources (32.1%) (Table 2).

**Table 2:** Sociodemographic characteristics of the participants (*n* = 106).

Variables	Frequency	Percentage
Age (Years)		
18–24	18	17
25–31	16	15.1
32–38	18	17
39–45	19	17.9
46–52	13	12.3
53–59	8	7.5
60–66	9	8.5
≥ 67	5	4.7
Gender		
Male	55	51.9
Female	51	48.1
Marital status		
Single	21	19.8
Married	77	72.6
widow\er	8	7.5
Educational level		
Illiterate	29	27.4
Primary school	23	21.7
Secondary school	34	32.1
Diploma	11	10.4
Bachelor+	9	8.5
Occupation		
Government employer	27	25.5
Self‐employed	42	39.6
Housewife	46	24.5
Student	4	3.8
Retired	7	6.6
Residence area		
Urban	65	61.3
Suburban	28	26.4
Rural	13	12.3
Financial status		
Sufficient	65	61.3
Barely sufficient	25	23.6
Insufficient	16	15.1
What's the client source of information about TBSs		
Relatives	60	56.6
Friends	10	9.4
Social media	2	1.9
Other	34	32.1

### 3.3 Injury and Treatment Characteristics

**Figure 1:**
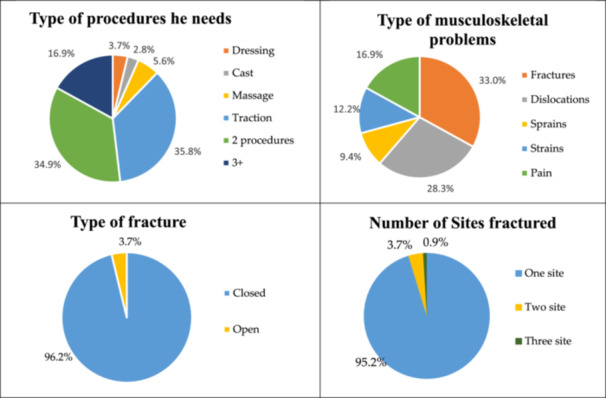
Distribution of the study sample according to certain medical problems (*n* = 106).

The analysis of injury patterns revealed that the most frequently affected anatomical sites included the upper extremities (hand: 22.1%, radius/ulna: 5.3%) and lower extremities (knee joint: 16.8%, foot: 19.5%) (Table 3). Additionally, vertebral injuries accounted for 16.8% of cases. The majority of participants (95.2%) had single‐site fractures, and 96.2% sought treatment for closed fractures (without open wounds). Among the treatment modalities utilized by TBSs, traction therapy was the most commonly performed procedure (35%), followed by massage and traditional joint adjustments (Figure 1 and Table 3).

**Table 3:** Frequencies and percentages for the site of fracture “multiple responses” (*n* = 106).

Sites of fracture‎	Frequency	Percentage
Upper extremities‐Shoulder joint	8	7.1
Upper extremities‐Humorous	7	6.2
Upper extremities‐Radius‎\Ulnar	6	5.3
Upper extremities‐Hand	25	22.1
Lower extremities‐Femur	3	2.7
Lower extremities‐Knee joint	19	16.8
Lower extremities‐Foot	22	19.5
Trunk‐Neck	2	1.8
Trunk‐Ribs	1	0.9
Trunk‐Vertebrae	19	16.8
Trunk‐Pelvic	1	0.9

### 3.4 Factors Influencing the Utilization of TBSs

The utilization of traditional bonesetters (TBSs) was primarily influenced by dissatisfaction with hospital‐based care rather than financial constraints. A majority of participants (67%) expressed dissatisfaction with hospital treatments, citing ineffective care and impersonal interactions with medical staff. Similarly, 67% reported negative experiences with hospital staff, highlighting issues related to poor communication and lack of empathy. While cost was assumed to be a major factor, only 25.5% of participants cited financial reasons for choosing TBSs, indicating that affordability played a lesser role than perceived treatment quality.

A substantial proportion (92.5%) of participants had positive past experiences with TBSs, reinforcing their trust in traditional methods, whereas 52% reported negative experiences with orthopedic specialists, further motivating their choice. Cultural beliefs were a factor for only 34% of participants, suggesting that faith in TBSs' efficacy rather than tradition alone influenced decisions. Additionally, fear of hospital procedures, particularly surgical interventions, was a significant factor, with 36.6% of participants preferring the perceived safety of non‐invasive treatments offered by TBSs (Table 4).

**Table 4:** Distribution of the study sample according to the factors that influenced the clients' decision to come to the TBSs (*n* = 106).

Variables	Categories	
	Yes	No
	*n* (%)	*n* (%)
Traditional bonesetters' fee	27 (25.5)	79 (74.5)
Not agree with the hospital's treatments	71 (67.0)	35 (33.0)
Not agree with hospital staff attitudes	71 (67.0)	35 (33.0)
Have good experience with TBSs	98 (92.5)	8 (7.5)
Cultural motivations	36 (34.0)	70 (66.0)
Fear of hospital procedures (surgery, amputation etc)	42 (36.6)	64 (60.4)
Have bad history with orthopedics	52 (49.1)	54 (50.9)

Table 5 presents the association between participants' sociodemographic characteristics, key factors influencing their decision to seek treatment from traditional bonesetters (TBSs), and their perceptions toward TBSs. The mean perception score was 4.80 ± 1.40, with a cutoff point of 5 used to categorize participant perceptions. Scores of 4 and lower indicated a negative perception, a score of 5 reflected a neutral perception, and scores of 6 or higher signified a positive perception toward TBSs. A positive perception of TBSs was significantly associated with several factors. Participants residing in suburban areas were more likely to perceive TBSs favorably (*p* = 0.037). Additionally, participants who disagreed with hospital treatments (*p* < 0.001) and hospital staff attitudes (*p* < 0.001) demonstrated a significantly higher likelihood of favoring TBSs. Furthermore, participants who had previously positive experiences with TBSs (*p* = 0.005) and those with a negative history with orthopedic care (*p* = 0.004) were also more inclined to view TBSs positively (Table 5).

**Table 5:** Association between some of the baseline sociodemographic characteristics and factors that influenced the clients' decision to come to the TBS with the perceptions of clients about TBSs (*n* = 106).

Variables		Perception levels			*χ* ^2^	*p* value
		Negative	Neutral	Positive		
		*n* (%)	*n* (%)	*n* (%)		
Age (Years)	18–24	4 (22.2)	7 (38.9)	7 (38.9)	19.33	0.152
	25–31	4 (25.0)	6 (37.5)	6 (37.5)		
	32–38	4 (22.2)	9 (50.0)	5 (27.8)		
	39–45	8 (42.1)	5 (26.3)	6 (31.6)		
	46–52	5 (38.5)	3 (23.0)	5 (38.5)		
	53–59	5 (62.5)	2 (25.0)	1 (12.5)		
	60–66	3 (33.3)	0 (0.0)	6 (66.7)		
	≥ 67	4 (80.0)	0 (0.0)	1 (20.0)		
Gender	Male	22 (40.0)	16 (29.1)	17 (30.9)	1.41	0.492
	Female	15 (29.4)	16 (31.4)	20 (39.2)		
Level of education	Illiterate	8 (27.6)	6 (20.7)	15 (51.7)	6.37	0.605
	Primary school	8 (34.8)	9 (39.1)	6 (26.1)		
	Secondary school	13 (38.2)	10 (29.4)	11 (32.4)		
	Diploma	5 (45.4)	4 (36.4)	2 (18.2)		
	Bachelor+	3 (33.3)	3 (33.3)	3 (33.4)		
Residence area	Urban	27 (41.5)	21 (32.3)	17 (26.2)	10.18	0.037*
	Suburban	7 (25.0)	5 (17.9)	16 (57.1)		
	Rural	3 (23.0)	6 (46.2)	4 (30.8)		
Financial status	Sufficient	21 (32.3)	24 (36.9)	20 (30.8)	5.44	0.245
	Barely sufficient	11 (44.0)	3 (12.0)	11 (44.0)		
	Insufficient	5 (31.3)	5 (31.3)	6 (37.4)		
Traditional bonesetter's fee	Yes	10 (37.1)	8 (29.6)	9 (33.3)	0.07	0.962
	No	27 (34.2)	24 (30.4)	28 (35.4)		
Not agree with the hospital's treatments	Yes	14 (19.7)	25 (35.2)	32 (45.1)	22.37	< 0.001*
	No	23 (65.7)	7 (20.0)	5 (14.3)		
Not agree with hospital staff attitudes	Yes	13 (18.3)	26 (36.6)	32 (45.1)	26.27	< 0.001*
	No	24 (68.6)	6 (17.1)	5 (14.3)		
Have good experience with TBSs	Yes	30 (30.6)	32 (32.7)	36 (36.7)	10.71	0.005*
	No	7 (87.5)	0 (0.0)	1 (12.5)		
Cultural motivations	Yes	14 (38.9)	8 (22.2)	14 (38.9)	1.64	0.440
	No	23 (32.9)	24 (34.2)	23 (32.9)		
Fear of hospital procedures	Yes	10 (23.8)	15 (35.7)	17 (40.5)	3.77	0.151
	No	27 (42.1)	17 (26.6)	20 (31.3)		
Have bad history with orthopedics	Yes	10 (19.2)	19 (36.6)	23 (44.2)	11.09	0.004*
	No	27 (50.0)	13 (24.1)	14 (25.9)		

* Significant.

### 3.5 Perceptions Toward TBSs

Participants' perceptions of TBSs varied, with many expressing strong confidence in their services. Approximately 74.5% of respondents believed TBSs were more reliable than orthopedic specialists, largely due to the personalized care and attentiveness they provided. Similarly, 71.7% perceived TBSs as treating patients with greater respect and empathy compared to hospital staff. The belief in TBS competence was prevalent, with 56.6% of participants considering them more skilled than formally trained orthopedic doctors.

Concerns about hospital‐based treatments further reinforced trust in TBSs, as 67.9% of participants viewed TBS treatments as having fewer side effects. Despite these strong positive perceptions, many respondents initially sought medical attention at hospitals before turning to TBSs. In fact, 63.2% of participants reported first visiting a hospital before consulting a TBS, indicating that dissatisfaction with modern healthcare often prompted the transition. Additionally, 50.9% of participants supported integrating TBSs into formal healthcare systems, reflecting a divided stance on the role of traditional healers in modern medicine (Table 6).

**Table 6:** Distribution of the study sample according to the perception of clients about TBSs (*n* = 106).

Items	Subscales	
	Yes	No
	*n* (%)	*n* (%)
He\she has a greater faith in TBSs	79 (74.5)	27 (25.5)
TSBs are more competent than orthopedics	60 (56.6)	46 (43.4)
He deals with clients more respectively	76 (71.7)	30 (28.3)
He believes that it has fewer side effects in contrast to medical treatments	72 (67.9)	34 (32.1)
At first, he went to the hospital	67 (63.2)	39 (36.8)
He was brought against his will	5 (4.7)	101 (95.3)
Believes in cooperation between orthopedics and TBSs	54 (50.9)	52 (49.1)

### 3.6 Levels of Perception Toward TBSs

Perceptions toward TBSs were almost evenly split, with 34.9% of participants holding positive views, citing successful past treatments, trust in traditional methods, and dissatisfaction with hospitals. An equal proportion (34.9%) had negative perceptions, raising concerns over the lack of formal training, risk of complications, and absence of scientific validation in TBS practices.

**Figure 2:**
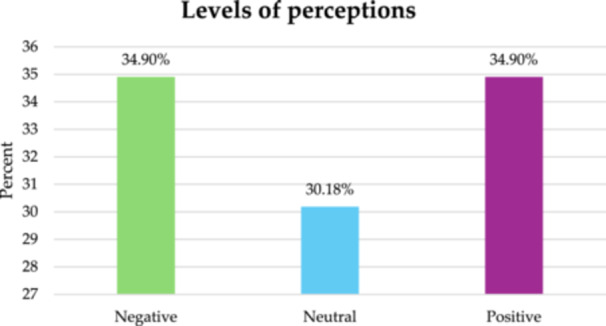
Percentages of the levels of perceptions in study sample (*n* = 106).

A significant proportion (30.2%) remained neutral, reflecting uncertainty about the efficacy of TBSs. These participants often relied on mixed personal experiences or incomplete information, contributing to indecision. The division of perspectives highlights the complexity of healthcare choices, where perceptions are influenced by treatment experiences, economic considerations, and trust in medical institutions (Figure 2).

### 3.7 Predictors of Positive Perceptions Toward TBSs

Multivariate logistic regression analysis identified dissatisfaction with hospital staff attitudes as the strongest predictor of positive perceptions toward TBSs (aOR: 0.04; 95% CI: 0.07–0.23; *p* < 0.001). Participants who reported poor communication and neglect in hospitals were significantly more likely to view TBSs favorably. Additionally, fear of hospital procedures, particularly surgical interventions, increased the likelihood of preferring TBSs (aOR: 0.13; 95% CI: 0.02–0.85; *p* = 0.033).

Residential location also played a role, with suburban residents more likely to have positive perceptions of TBSs compared to urban dwellers (*p* = 0.037). This may reflect limited access to specialized healthcare services in suburban areas, increasing reliance on traditional treatments. However, demographic factors such as age, gender, and financial status were not significantly associated with perceptions toward TBSs, suggesting that healthcare experiences and provider trust were more influential in shaping attitudes than socioeconomic characteristics (Table 7).

**Table 7:** The regression analysis results for determining predictive factors perception of clients about TBSs of the participants (*n* = 106).

Variables	Univariate				Multivariate			
	Positive perception				Positive perception			
	OR	95% CI		*p*	AOR	95% CI		*p*
Age (18‐24 as ref.)								
25–31	7.00	0.56	86.3	0.129	14.56	0.26	84.64	0.191
32–38	6.00	0.47	75.34	0.165	9.01	0.16	45.27	0.282
39–45	5.00	0.38	64.38	0.217	7.21	0.13	39.44	0.335
46–52	3.00	0.26	34.19	0.376	2.97	0.06	10.65	0.572
53–59	4.00	0.32	49.59	0.280	3.81	0.06	26.27	0.515
60–66	0.80	0.03	17.19	0.887	1.83	0.01	28.36	0.804
≥ 67	8.00	0.59	106.93	0.116	20.94	0.24	118.77	0.182
Gender (Male as ref.)								
Female	3.76	0.46	30.49	0.214	0.85	0.19	3.81	0.840
Level of education (Illiterate as ref.)								
Primary school	11.87	0.17	821.18	0.252	—	—	—	—
Secondary school	1.03	0.02	41.25	0.987	—	—	—	—
Diploma	0.29	0.01	8.03	0.470	—	—	—	—
Bachelor+	0.45	0.08	27.35	0.709	—	—	—	—
Residence area (Urban as ref.)								
Suburban	0.03	0.01	0.89	0.043	0.10	0.09	1.27	0.077
Rural	0.56	0.02	11.34	0.711	1.22	0.10	13.68	0.872
Financial status (Sufficient as ref.)								
Barely sufficient	6.09	0.42	87.26	0.183	3.23	0.43	24.09	0.251
Insufficient	3.04	0.27	34.14	0.366	2.24	0.28	17.97	0.445
Traditional bonesetter's fee (Yes as ref.)								
No	1.32	0.16	10.53	0.791	—	—	—	—
Not agree with the hospital's treatments (Yes as ref.)								
No	1.75	0.08	36.96	0.718	—	—	—	—
Not agree with hospital staff attitudes (Yes as ref.)								
No	0.09	0.01	0.29	0.008*	0.04	0.07	0.23	< 0.001*
Have good experience with TBSs (Yes as ref.)								
No	0.03	0.01	2.42	0.123	0.12	0.07	2.18	0.154
Cultural motivations (Yes as ref.)								
No	2.13	0.33	13.43	0.419	2.58	0.51	13.008	0.249
Fear of hospital procedures (Yes as ref.)								
No	0.11	0.01	0.98	0.048	0.13	0.02	0.85	0.033*
Have bad history with orthopedics (Yes as ref.)								
No	0.24	0.03	1.53	0.134	0.30	0.07	1.22	0.09

*Significant.

## 4 Discussion

Although traditional bonesetting has been addressed extensively in previous literature, the present study provides novel insights into the socio‐behavioral drivers of TBS utilization in the Kurdish context. It is important to note that treatment by non‐medical personnel and traditional healers is contrary to international medical guidelines due to the risk of severe complications, unsafe practices, and non‐sterile techniques documented in multiple regions [11, 12]. As in many developing settings, this constitutes a persistent public health challenge that demands coordinated interventions by policymakers, healthcare providers, and community leaders to mitigate associated risks.

The present study results showed that the TBSs that participated in the present study acquired the knowledge by learning through personal efforts or from their father, none of them graduated from an academic institution or even there was no interaction between them and orthopedics. They know a little about human anatomy, physiology, complications and investigations as is the case with all traditional bonesetters [18], however, both of them request X‐rays and do not work with clients with chronic diseases, which seems to be why they haven't faced any problems or complications. One of the TBSs said it could locate the fracture manually, a process in which the boneset looks for bone cracks under the skin and muscles or waits for the client's reaction to a gentle touch, that's a strategy used by many experienced traditional bonesetters [19]. According to the TBSs, they do not receive money for their work however, by looking at the results the majority of participants' financial status was sufficient, it can be derived from the results that clients have greater faith in TSBs than orthopedics and they have best perceptions toward TSBs, most of them reported that TSBs deals with clients respectively and honestly, therefore by observing the results it becomes clear that the main factor that clients select TSBs is not economical aspects, transcultural beliefs or religious motivations rather, it stems from believes in the work of the TBSs [20].

The results of the present study showed that the majority of participants were at the age of production at this age with fracture, people are more likely to have accidents, especially traffic accidents as an etiology of fracture [12].

When comparing the present findings with studies from other regions, notable similarities and differences emerge. For instance, in Nigeria and Ghana, dissatisfaction with hospital staff attitudes and perceived procedural risks have also been cited as major drivers for choosing TBSs [3, 4], aligning closely with the patterns observed in Kurdistan. However, unlike reports from rural African settings where economic constraints and cultural traditions are dominant motivators ([8]; Onyemaechi [6]), our findings suggest that belief in the competency and respectful care of TBSs outweighs financial or cultural drivers. This difference may reflect the relatively better economic status and healthcare access in certain Kurdish urban areas, where dissatisfaction stems less from scarcity and more from interpersonal and trust‐related issues. These insights underscore the need for culturally adapted interventions—such as integrating communication skills and patient engagement strategies into hospital training programs—that address local perceptions while remaining aligned with evidence‐based orthopedic standards. An instructive example from other countries is the gradual replacement or transformation of traditional bonesetters into formally trained and licensed mid‐level practitioners, often referred to as “orthopedic officers” or “orthopedic assistants.” These professionals receive structured education, operate strictly under the supervision of orthopedic surgeons, and adhere to recognized treatment protocols without employing unvalidated methods. Introducing such a cadre in Kurdistan could preserve the community trust and accessibility advantages of TBSs while ensuring compliance with evidence‐based orthopedic care and minimizing the risks associated with traditional, unregulated practices.

On the other hand, results showed that there was a significant association between residential areas and perceptions about TBSs, whereas most participants were urban dwellers. Sina et al. in 2015, reported that most of TSBs patients are rural dwellers, and urban residents are less likely to go to TSBs [20]. Theoretically, healthcare services are more available in urban areas than in rural, so studies often find that rural residents use traditional medicine more. However, when the issue is related to people's beliefs and people's perceptions, it crosses medical boundaries, the result is that people use more TBSs despite the availability of orthopedics. By looking at the results show that the participants expressed that they believed more in TBSs, reporting that they were more competent than orthopedics and they are not satisfied with the healthcare services available in hospitals [3]. Most of them went to the hospital first and then returned to TBSs. However, they did not hide that they want to have coordination between TBSs and orthopedics, whereas other studies reported the contrasting results (Onyemaechi [21]). Finally, fear of hospital procedures was one of the most common causes and could predict perceptions about TBSs [20].

The findings also highlight underlying systemic challenges within the local healthcare environment. While “not agreeing with hospital staff attitudes” emerged as a significant factor, this should be interpreted as a symptom of deeper structural issues, potentially including inadequate provider–patient communication, insufficient counseling on treatment options, negative interpersonal behaviors, perceived medical malpractice, or gaps in clinical training. These elements warrant further investigation, as they may collectively undermine patient trust and prompt individuals to bypass formal healthcare in favor of traditional practitioners. Addressing these issues through targeted hospital quality improvement programs, enhanced communication skills training for staff, and transparent complaint‐resolution mechanisms may help rebuild patient confidence and reduce reliance on TBSs.

## 5 Limitations

This study has several limitations that should be considered. The use of non‐probability purposive sampling may limit the generalizability of findings, suggesting that future studies should employ randomized sampling techniques for broader representation [22]. Additionally, the reliance on self‐reported data introduces the potential for recall and social desirability bias, which may affect the accuracy of participants' responses regarding their experiences with TBSs and hospital‐based care. To mitigate this, future research should incorporate medical records or direct observational methods to validate findings. Another limitation is the relatively small sample size [22, 23]. This may have reduced the statistical power of the study, particularly in multivariate regression analysis. Increasing the sample size in future research would enhance the robustness of predictive modeling and allow for more comprehensive subgroup analysis. Finally, the study did not assess long‐term patient outcomes, making it difficult to determine the efficacy and safety of TBS treatments over time. Longitudinal studies are recommended to evaluate treatment effectiveness, complication rates, and potential long‐term risks associated with TBS interventions. Finally, although several determinants of TBS utilization were examined, some potentially important confounding variables—such as patients' health literacy, accessibility of specialized orthopedic services, and perceived convenience—were not fully explored. Including these in future models could improve the accuracy and completeness of findings.

## 6 Conclusion

Factors that influence people to select traditional bonesetters in Kurdistan Northern Iraq can be combined with not agreeing with hospital staff attitudes toward neuro and musculoskeletal problems, not agreeing with the hospital treatments and having a bad history with orthopedics. Having a positive perspective toward bonesetters stems more from having good experience with TBSs than from cultural motivations and TBSs fees. While the health system in this region is in the process of developing, and there are different specialties in the healthcare system, however, TBSs cannot be ignored. The results of the present study are not conclusive but can be a basis for future studies. Based on these findings, we recommend that future research specifically investigate deficiencies in medical practice within local hospitals, including provider communication, procedural transparency, and adherence to treatment protocols. Interventions aimed at improving the quality of care—through staff training, supervision, and infrastructure support—should be prioritized to align with international standards. Additionally, targeted health education programs for patients and their families are essential to enhance awareness of safe, evidence‐based orthopedic treatment options and to empower informed healthcare decision‐making.

## Author Contributions

**Salar Omer Abdulqadir:** conceptualization, methodology, writing – original draft, writing – review and editing, data curation, formal analysis, resources, investigation, visualization, project administration. **Rukhsar Muhammad Omar:** conceptualization, investigation, writing – original draft, visualization, validation, methodology, resources, data curation, project administration, writing – review and editing. **Sirwan Khalid Ahmed:** conceptualization, investigation, writing – original draft, supervision, data curation, resources, software, formal analysis, methodology, writing – review and editing, validation, visualization. **Moustaq Karim Khan Rony:** conceptualization, writing – original draft, writing – review and editing, methodology, formal analysis, supervision, validation, visualization, investigation.

## Funding

The authors received no specific funding for this work.

## Ethics Statement

This study was conducted in accordance with the Institutional Research Ethics Board and the Declaration of Helsinki to uphold ethical integrity and participant rights. The research received ethical approval from the Scientific Review Committee of the University of Raparin, College of Nursing (Ref: 7/29/3721).

## Conflicts of Interest

The authors emphasize that no one has an interest or competition to affect this work and the results of the present study.

## Transparency Statement

The lead author Sirwan Khalid Ahmed, Moustaq Karim Khan Rony affirms that this manuscript is an honest, accurate, and transparent account of the study being reported; that no important aspects of the study have been omitted; and that any discrepancies from the study as planned (and, if relevant, registered) have been explained.

## Data Availability

The data that support the findings of this study are available from the corresponding author upon reasonable request.
